# Crisis support in practice

**DOI:** 10.1192/j.eurpsy.2025.213

**Published:** 2025-08-26

**Authors:** J. Nordholm

**Affiliations:** Affective Disorders, Sahlgrenska University Hospital, Gothenburg, Sweden

## Abstract

**Abstract:**

An experienced practitioner describes the fundamentals of crisis support and Psychological First Aid – the knowledge base for the ongoing work in Västra Götalandsregionen, Sweden, using VR to practice crisis support. How is crisis support defined and what is the difference between crisis support and psychological treatment? What is the aim of crisis support? The presentation includes current knowledge about how humans react in a crisis, in the acute phase as well is in the longer term. What are the guidelines for providing Psychological First Aid and how can we implement them in practice?

**Disclosure of Interest:**

None Declared

